# Action of SNAIL1 in Cardiac Myofibroblasts Is Important for Cardiac Fibrosis following Hypoxic Injury

**DOI:** 10.1371/journal.pone.0162636

**Published:** 2016-10-05

**Authors:** Hirak Biswas, Gregory D. Longmore

**Affiliations:** 1 Department of Medicine, Washington University, St. Louis, MO, 63110, United States of America; 2 Department of Cell Biology and Physiology, Washington University, St. Louis, MO, 63110, United States of America; 3 ICCE Institute, Washington University, St. Louis, MO, 63110, United States of America; Centre National de la Recherche Scientifique, FRANCE

## Abstract

Hypoxic injury to the heart results in cardiac fibrosis that leads to cardiac dysfunction and heart failure. SNAIL1 is a zinc finger transcription factor implicated in fibrosis following organ injury and cancer. To determine if the action of SNAIL1 contributed to cardiac fibrosis following hypoxic injury, we used an endogenous SNAIL1 bioluminescence reporter mice, and SNAIL1 knockout mouse models. Here we report that SNAIL1 expression is upregulated in the infarcted heart, especially in the myofibroblasts. Utilizing primary cardiac fibroblasts in ex vivo cultures we find that pro-fibrotic factors and collagen I increase SNAIL1 protein level. SNAIL1 is required in cardiac fibroblasts for the adoption of myofibroblast fate, collagen I expression and expression of fibrosis-related genes. Taken together this data suggests that SNAIL1 expression is induced in the cardiac fibroblasts after hypoxic injury and contributes to myofibroblast phenotype and a fibrotic scar formation. Resultant collagen deposition in the scar can maintain elevated SNAIL1 expression in the myofibroblasts and help propagate fibrosis.

## Introduction

Hypoxic (ischemic) injury to cells and organs, such as occurs to the heart during myocardial infarction due to artery occlusion, has been viewed as a wound healing process[1]. Hypoxia causes death of cardiac cells and these dying cells release inflammatory mediators that result in an inflammatory response in the injured region. This inflammatory response sets up a wound healing cascade which ultimately deposits a fibrous scar tissue in the infarcted region. The scar consists of deposited extracellular matrix (ECM), and is the attempt to prevent rupture of the heart[1]. When the scarring reaction is persistent this can lead to excess deposition of extracellular matrix (ECM) and cardiac fibrosis, which results in stiffening of the heart, reduced cardiac output (ventricle dysfunction), and heart failure[1]. Therefore, understanding of the mechanisms for and regulation of cardiac fibrosis following injury could result in the development of anti-fibrotic therapies to improve quality of life after myocardial infarction.

Within the heart, fibroblasts are the major cell type responsible for generating the fibrotic reaction after ischemic injury[2]. In term of numbers, cardiac fibroblasts make up the majority of the cells in the heart, even outnumbering cardiomyocytes[3]. In response to ischemic injury, the number of cardiac fibroblasts at the injury site increase and become activated to myofibroblasts[2]. The increased number of myofibroblasts in the scar area can derive from various sources that include: proliferation of resident cardiac fibroblasts, endothelial to mesenchymal transition of endothelial cells, circulating fibrocytes, pericytes, inflammatory cells and bone marrow derived mesenchymal progenitor cells[2]. These activated fibroblasts, or myofibroblasts, express the markers alpha smooth muscle actin (αSMA) and periostin and secrete pro-fibrotic and pro-inflammatory cytokines as well as ECM proteins and ECM modifying enzymes. The deposited ECM is mainly comprised of fibrillar collagens (collagens I and III). Periostin, secreted by myofibroblasts, regulates the alignment of the collagen I fibrils[4] while secreted enzymes that cross-link collagen fibers (e.g., lysyl oxidases) can increase tissue stiffness[5].

A number of transcriptional regulators have been implicated as contributing to fibroblast to myofibroblast phenotypic switch. Their expression is activated by the inflammatory cytokines released after hypoxic injury. For example, the serum response factors (SRF) Myocardin Related Transcription Factor A/B (MRTFA/B)[6]. Another critical mesenchymal cell transcriptional regulator is SNAIL1, and SNAIL1 has been shown to be important for organ fibrosis that develops following injury to the liver[7] and kidney[8], as well as the fibrosis associated with some cancers[9],[10]. Increased SNAIL1 levels in endothelial cells following ischemia reperfusion cardiac injury has been reported and is associated with secretion of connective tissue growth factor (CTGF), which in turn activates neighboring cardiac fibroblasts to myofibroblasts [11]. Whether SNAIL1 is necessary for cardiac fibrosis following ischemic injury, and if so in what cells in the heart and how, has not been determined.

SNAIL1 is a critical mesenchymal cell fate regulator and a zinc finger transcriptional repressor, although a recent study suggests that under certain conditions and in the presence of specific co-factors it can act as a transcriptional activator[12]. SNAIL1 protein level and function is regulated at the transcriptional and post-transcriptional levels by various biochemical and mechanical extracellular stimuli that can be present in the hypoxia-injured heart. These include: TGFβ, PDGF, hypoxia and increased ECM stiffness. Using a SNAIL1 reporter mouse and immunohistochemical analyses we find that SNAIL1 levels are increased in heart fibroblasts following hypoxic injury. Through genetic manipulation of SNAIL1 expression in primary heart fibroblast cells we find that SNAIL1 is critical for the formation and function of myofibroblasts and their fibrotic response following TGFβ stimulation.

## Materials and Methods

### Mice and animal husbandry

All mice were housed and experiments performed according to institutional guidelines. Production of SNAIL1-CBR/+ mice has been previously described[13]. SNAIL1^*f/f*^ mice were provided by S. Weiss (U. Michigan)[14]. All mice were on mixed genetic backgrounds. Experiments were carried out on 8–10 week old mice. All mice were used in compliance with the Washington University Institutional Animal Care and Use Committee under protocol #20150145. All mouse experiments were reviewed and approved by the Washington University Institutional Animal Care and Use Committee under protocol #20150145. Approximately 20 SNAIL1-CBR/+ mice and 20 SNAIL1^*f/f*^ mice were used for these experiments. Five mice were kept per cage with 12 hour light / 12 hour dark cycle and standard rodent chow and water available *ad libitum*. The mice were monitored everyday.

### Mouse surgeries and tissue processing

#### Left anterior descending artery (LAD) occlusion and closed chest ischemia

Reperfusion (I/R) surgeries were performed at the Mouse Cardiovascular Phenotyping Core at Washington University, in compliance with the Washington University Institutional Animal Care and Use Committee under protocol #20150145. For Closed-Chest I/R surgery, the mice were surgically prepped and ventilated. The mice were taped to an ECG board (lead II) to measure S-T segment elevations during ischemia and reperfusion. After a midline incision and small “non-rib cutting” thoracotomy, the pericardium was gently dissected to visualize the coronary anatomy. An 8–0 polypropolene suture with a U-shaped tapered needle was passed under the LAD at a consistent level on the heart directly underneath the LA. The needle was then cut from the suture and the two ends of the 8–0 suture was threaded through a 0.5mm piece of PE-10 tubing that had been previously soaked for 24 hours in 100% ethanol. The tubing formed a loose snare around the LAD. Each end of the suture was then threaded through the end of a size 3 Kalt suture needle and exteriorized through each side of the chestwall. The chest was closed closed with interrupted stitches. The ends of the exteriorized 8–0 suture was tucked under the skin and the skin closed. The mice were removed from the respirator, kept warm and allowed to recover to full consciousness. After a recovery period of 1 week after initial instrumentation, the animals were reanesthetized under isoflurane (1.5% maintenance) but not mechanically ventilated and only the skin above the chest wall was reopened. The 8–0 suture ends were cleared of all debris and carefully secured in small hemostats. Ligation of the LAD for 90 minutes was accomplished by gently pulling the hemostats apart and anchoring them until the S-T segment elevation appeared on the EKG. The EKG was constantly monitored throughout the occlusion period to ensure persistent ischemia. At the end of the 90 minutes, reperfusion was accomplished by releasing the hemostats, cutting the suture close to the chest wall, and releasing the tension. Reperfusion was confirmed by resolution of the S-T segment elevation. The skin was closed with suture and the animal was allowed to recover on a warmer for 1–7 days.

Permanent LAD occlusion surgery.

Mice were anesthetized with Ketamine/Xylazine (100/10 mg/kg) and surgically prepped and ventilated on a heated magnetic stainless steel surgical board. After a midline incision and small “non-rib cutting” thoracotomy, the chest wall was retracted to better expose the left ventricle and the left main coronary artery system. The left anterior descending branch of the left coronary artery was then ligated with an 8–0 silk suture. This occlusion will be accomplished by passing a tapered needle modified to a U-shape, underneath the LAD at a consistent level on the heart directly underneath the left atrium and tying this suture directly over the vessel. Mice were then hyperventilated at 150 beats/min until the chest was closed by purse string suture. The surgical incision was closed in two layers with an interrupted suture pattern. The animals were allowed to recover and kept warm on a heating pad throughout the procedure until extubation, return to sternal position, and normal activity. The mice were monitored twice daily for 7 days.

Hearts from the infarcted mice were harvested 7 days post infarction. Hearts were rinsed in PBS and placed in 10% neutral buffered formalin overnight at room temperature. The hearts were cut using a zivic heart slicer into infarcted region (distal to ligation) and remote region (proximal to ligation). The hearts sections were either placed in 70% ethanol for paraffin embedding or cryoprotected in 30% sucrose for embedding in Optimal Cutting Temperature (OCT) compound. For mRNA isolation, the left ventricle was dissected under a dissecting microscope and mRNA was extracted using a Qiagen mini RNA kit.

### Cardiac fibroblast isolation and cell culture

Cardiac fibroblasts were isolated according to a modified cardiomyocyte isolation protocol. Hearts were obtained from mice after anesthetizing with isofluorane followed by cervical dislocation. The hearts were excised, placed in sterile PBS. In a tissue culture hood, hearts were finely minced with sterile scissors and transferred into a 50 ml falcon tube with 9 mL of Wiittenberg Isolation Medium [NaCl (116 mM), KCl (5.4mM), MgCl2 (6.7mM), glucose (12mM), glutamine (2mM), NaHCO_3_ (3.5mM), KH_2_PO_4_ (1.5mM), Na_2_HCO_3_ (1mM), HEPES (21mM), commercial vitamin solution (1X), commercial animo acid solution (1X)] supplanted with trypsin (1X) and collagenase II (0.8 mg/mL). The minced tissue was placed on a rotator in a 37°C incubator for 15 minutes. The tubes were spun down for 5 minutes and the supernatant (extract 1) was discarded. The extraction process was repeated 4 times with the remaining tissue and the supernatants (extract 2–5) were pooled, spun down and plated in p100 tissue culture dishes in DMEM/F12 media with 10% Fetal Bovine Serum and 1% penicillin–streptomycin. The media was changed after 6 hours to allow cells to adhere and wash away the debris. Media was replaced every other day until cells were confluent (~5 days). Fibroblast identity was verified by vimentin and DDR2 expression.

### Bioluminescence imaging

For live animal and *ex-vivo* heart imaging, the mice were injected with d-luciferin 10 minutes before imaging, anesthetized using isoflurane and imaged with the IVIS-100 instrument.

For live cell imaging, 8x10^3^ SNAIL1-CBR/+ primary cardiac fibroblasts (passage 2 or 3) were plated in 96 well black well plates. After 20 hours, cells were washed with PBS, and serum free, phenol red-free medium added. After 4 hours of serum starvation, d-luciferin was added along with one of the following: TGFβ (2ng/mL), PDGF (10ng/mL), Angiotensin II (1 micromol/L), CoCl_2_ (400uM). The plate was imaged every 15 minutes for 4 hours with IVIS 100 instrument at 37°C with ~5% O_2_ flow the imaging chamber. For collagen I stimulation, the cells were serum starved and then plated on tissue culture plates coated with 60 uL of 1–4 mg/mL collagen I gel and imaged as described before.

### SNAIL1 gene deletion in cardiac fibroblasts

To delete the SNAIL1 gene, 1x10^6^ SNAIL1^*f/f*^; ROSA-LSL-tdTomato cardiac fibroblasts were infected with either Adeno-LacZ (control, denoted CTL) or Adeno-Cre (experimental, denoted + Cre) viruses at an MOI of 50. The virus was removed after 4 hours, cells washed and used 48 hours post infection. The rate of infection was >98% as determined by tdTomato expression. SNAIL1 gene deletion was verified by qPCR and western blotting. Cells were freshly infected for each experiment.

### qPCR Analyses

500 ng of mRNA was used to synthesize cDNA using the Invitrogen Superscript II kit. qPCR was performed using 1uL of cDNA in a 20 uL reaction using the SyBr green reagents (Applied Biosystems) on StepOnePlus instrument (Applied Biosystems). The cT values were normalized to GAPDH and the fold change was calculated using the 2^-ddCT^ method.

### Western blotting

Western blotting was done according to standard protocols. 50ug protein was loaded in each lane. The following antibodies were used: SNAIL1 (1:100 Cell Signaling), Periostin (1:500, gift from Dr. Russell Norris, MUSC), β tubulin (1:10,000 Sigma), DDR2 (1:500, Cell Signaling) ERK (1:1000, Cell Signaling) phospho-ERK (1:1000, Cell Signaling), AKT(1:1000, Cell Signaling), phospho-AKT(1:1000, Cell Signaling), STAT1 (1:1000, Cell Signaling), phospho-STAT1 (1:1000, Cell Signaling).

### Ex vivo Matrix deposition and analysis

A modified scar-in-a-jar assay was used for matrix deposition by cardiac fibroblasts[15]. 1x10^5^ control or SNAIL1 knockout cells were plated on sterile 100mm coverslips overnight. The cells were then treated everyday with fresh media containing 50 ug/mL ascorbic acid for 7 days, supplemented with 10 ng/uL PDGF every other day. The cells were removed by treating with prewarmed extraction buffer for 3–5 minutes (25 mmol/L Tris-HCl, pH 7.4; 150 mmol/L sodium chloride; 0.5% Triton X-100; and 20 mmol/L ammonium hydroxide).

For collagen I immunofluorescence (1:100, BD bioscience antibody), the matrix was fixed with 4% paraformaldehyde for 10 minutes. For Sirius red staining cells were fixed in bouins fixative for 20 minutes and washed in tap water. Staining was then performed according to standard protocol[16]. Birefringence imaging was performed on the Sirius red stained sections under polarized light[17].

### Gel Contraction Assay

5x10^4^ CTL or Experimental (+Cre) cardiac fibroblasts were embedded in 2mg/mL of collagen I gel (Rat tail collagen 4mg/mL, 10%FBS and 23uL of NaOH /mL of collagen). 500uL of the cells-collagen was plated in 24 well plate in triplicate for each condition. The gel was solidified at 37°C for 30 minutes and the gel was released from the sidewall of the well by gently running a 10uL pitette tip. 1mL of DMEM/F12 media with 2% FBS and 1% Penicillin-Streptomycin was added on top. The gels were imaged at baseline and every 4 hours subsequently. The percent contraction was measured by comparing the initial image area to contracted image area by ImageJ software (surface area function). Data representative of 3 independent expriments.

### Histology and immunostaining

5 um sections of paraffin or OCT embedded hearts were utilized for Massons Trichrome Staining (KY034, Diagnostic BioSystems) and immunofluorescence. Immunofluorescence was performed according to standard protocol. The following antibodies were used: SNAIL1 (1:100, Cell Signaling), αSMA (1:300, Sigma), Periostin (1:100, Cell Signaling), CD31 (1:200, Abcam), CD45 (1:100, BD Biosciences), α-actinin (1:100, Abcam). For SNAIL1 immunofluorescence, antigen retrival was done in Nuclear Decloaker solution (CB911M, Biocare Medical) and TSA plus kit (Perkin Elmer) was used to amplify SNAIL1 signal.

### Image quantification

Sirius Red stained images and birefriengence images were analyzed with a custom image segmentation algorithm written in MatLab (The Mathworks, Inc.). Images were converted from the standard RGB color space to the CIE L*a*b* color space, which encodes perceived lightness and color differences well. Each pixel in the image was classified as positive (collagen I) or negative (background) based on its distance in CIE L*a*b* space to the closest member of an empirically determined set of positive and negative training pixels. Positive signal was quantified as the fraction of positive pixels out of the total number of pixels in an image.

### Statistics

Students t test was used to determine statistical significance. p values < 0.05 was considered statistically significant.

## Results

### SNAIL1 expression in the heart increases post myocardial infarction

**Fig 1:**
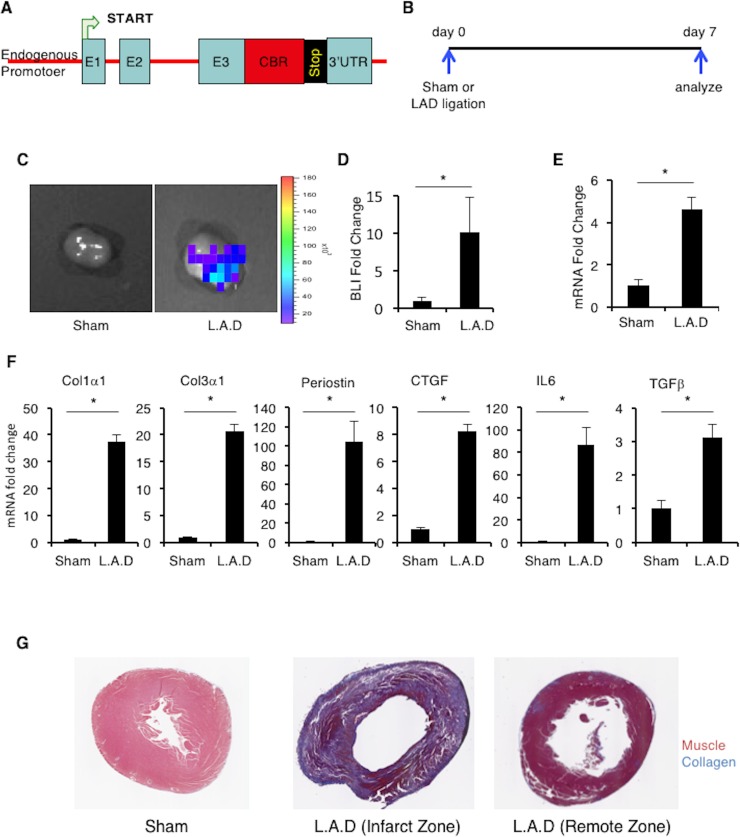
**SNAIL1 expression increases in mouse heart post LAD ligation. (A)** Schematic representation of the SNAIL1-CBR targeting construct. E1,E2,E3 are the three exons of SNAIL1-CBR is clic beetle red bioluminescent protein. **(B)** The timeline of the experiment. **(C)** SNAIL1-CBR bioluminescence signal in sham (n = 3) and infarcted hearts (n = 4) after Left Anterior Descending Artery Occlusion (LAD). **(D)** Quantification of biolumincence signal in (C). **(E)** Relative level of SNAIL1 mRNA in LAD ventricles, normalized to SNAIL1 mRNA from sham treated ventricles, as determined by Q-PCR. SNAIL1 mRNA level in sham treated hearts was arbitrarily set to 1. **(F)** Relative mRNA levels for select fibrogenic genes, as indicated, in LAD ventricles normalized to sham treated ventricles. Determined by Q-PCR. SNAIL1 mRNA level in sham treated hearts was arbitrarily set to 1. Representative of 3 hearts. **(G)** Representative images of trichrome staining for collagen in Sham and LAD sections. All experiments were repeated three times with three mice (minimum) in each group.

To determine whether SNAIL1 expression in the heart was induced in response to ischemic cardiac injury, we made use of a previously described SNAIL1-Click Beetle Red (CBR) fusion bioluminescence reporter mouse (Fig 1A)[18]. In this mouse the CBR bioluminescent enzyme was inserted into the SNAIL1 gene, in frame, downstream of the third (terminal) exon of SNAIL1 so as to generate a SNAIL1-CBR fusion allele. Through this design, SNAIL1-CBR expression is under regulation of the endogenous SNAIL1 promoter.

Heterozygote SNAIL1-CBR/+ mice did not express SNAIL1-CBR in the uninjured normal heart (S1A Fig) and no abnormal phenotype in any organ was observed[18]. To induce hypoxic cardiac injury, SNAIL1-CBR/+ mice were subjected to Left Anterior Descending (LAD) Artery ligation. Hearts were harvested and analyzed 7-days post-surgery (Fig 1B). In sham treated mice there was minimal SNAIL1-CBR bioluminescence detected, but in mice subjected to LAD ligation these was a 10-fold increase in SNAIL1-CBR bioluminescence signal (Fig 1C, quantified in Fig 1D). SNAIL1 mRNA levels also increased in the hearts of mice exposed to hypoxic injury, as determined by Q-PCR of tissue from the ventricular region of infarcted and sham surgery control hearts (Fig 1E). Fibrogenic gene expression (e.g., collagen I and III, Connective Tissue Growth Factor (CTGF), Interleukin 6 (IL6), Transforming Growth Factor beta (TGFβ) and periostin increased in the infarct region (Fig 1F). Increased SNAIL1-CBR level was associated with the presence of fibrosis in the infarct region (Fig 1G). We also subjected SNAIL1-CBR reporter mice to the ischemia-reperfusion (IR) injury model. SNAIL1-CBR bioluminescence, SNAIL1 mRNA, and fibrogenic gene mRNA level all increased in the IR model as well (S1C–S1H Fig).

In sum, following ischemic injury to the heart SNAIL1 mRNA and protein level increased in the infarcted zone.

### Myofibroblasts within the infarct zone express SNAIL1

**Fig 2:**
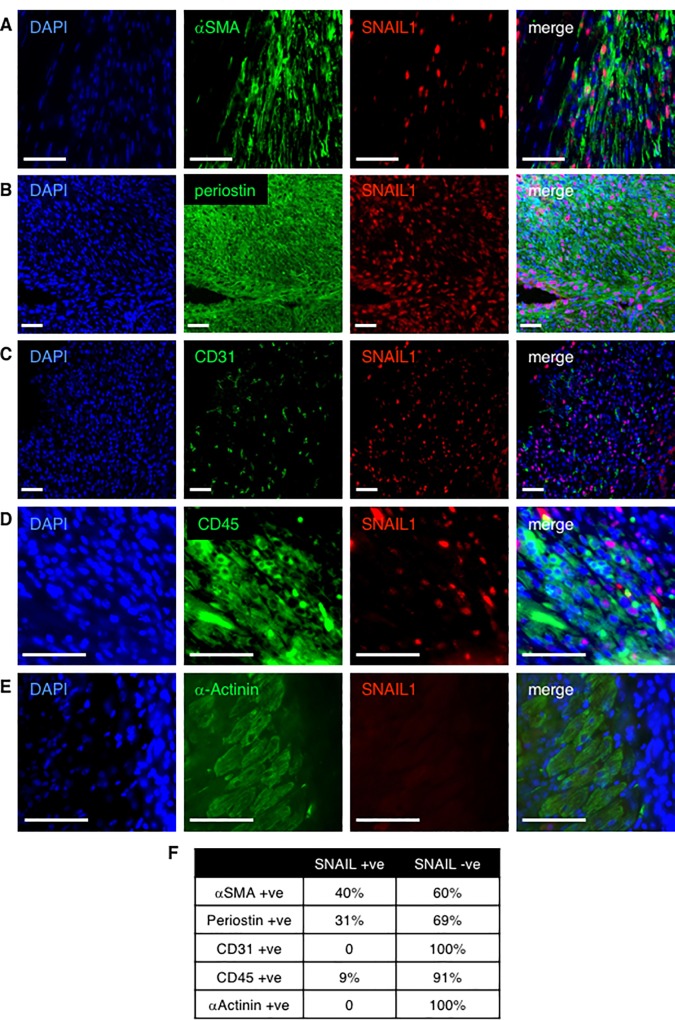
**SNAIL1 expression in infarct zone of the heart.** Immunofluoresecnce staining of SNAIL1 and cardiac cell type markers in the ventricular infarct zone of LAD surgery mice. Cardiac cell type markers: **(A)** αSMA (alpha smooth muscle actin)–myofibroblast marker **(B)** periostin–myofibroblast marker, **(C)** CD31 –endothelial cell marker, **(D)** CD45 –leukocyte cell marker, **(E)** α-actinin–cardiomyocyte marker. Scale bars: 50 microns. Infarcted hearts from six mice were analyzed. There were 4–5 images per cell type per heart. **(F)** Table quantifying marker staining in SNAIL1 positive and SNAIL1 negative cells within the infarcted heart.

To identify which cells within the infarct zone expressed SNAIL1, we performed co-immunostaining with different cardiac cell type markers and CD45 (infiltrating leukocytes). SNAIL1 staining was observed only in the infarct zone and not the non-injured, remote zone (Fig 2A–2E and S2 Fig). A significant number of αSMA (40%) and periostin (31%) positive myofibroblasts in the infract zone expressed SNAIL1 (Fig 2A and 2B, quantified in Fig 2F). A small proportion (<10%) of CD45 positive leukocytes expressed SNAIL1 (Fig 2D, quantified in Fig 2F). SNAIL1 expression was not detected in α-actinin positive cardiomyocytes within the infarct zone (Fig 2E) and contrary to previous reports[11] we did not observe SNAIL1 staining in CD31 positive cells within the infarct zone. In sum, these results indicated that following cardiac ischemic injury there was an increase in SNAIL1 protein and mRNA expression within the infarct zone, predominantly in activated myofibroblasts.

### Pro-fibrotic factors increase SNAIL1 expression in cardiac fibroblasts

**Fig 3:**
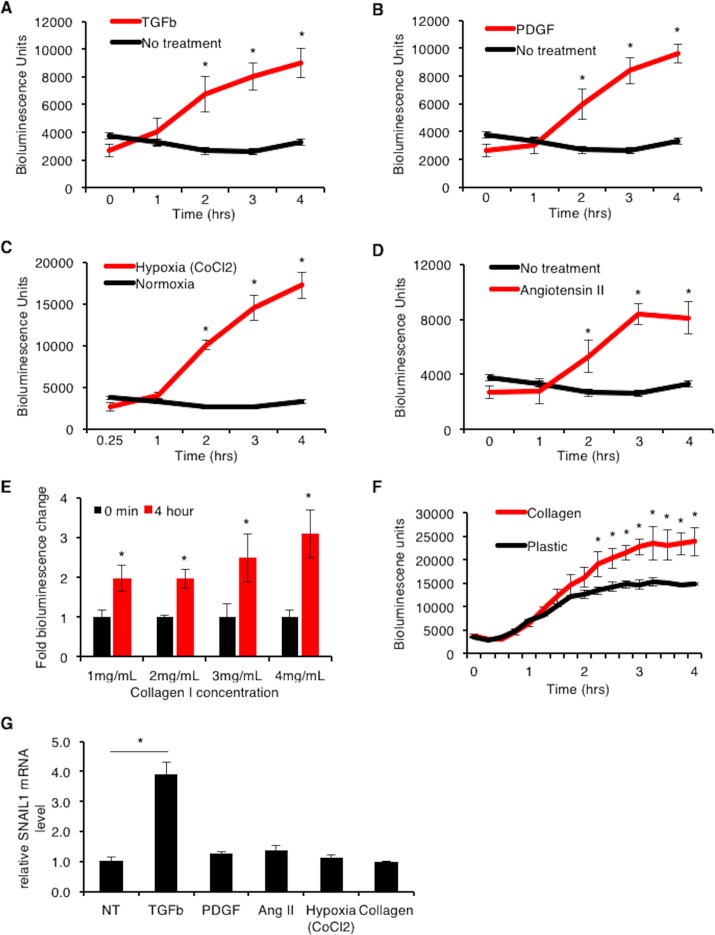
**Pro-fibrotic factors increase SNAIL1-CBR level in primary cardiac fibroblasts.** Primary fibroblasts were isolated from the hearts of un-infarcted, normal SNAIL1-CBR/+ mice. Bioluminescence intensity after treatment with TGFβ (2 ng/uL) **(A)**, PDGF (10 ng/uL) **(B)**, Hypoxia (400 uM CoCl2) **(C)** and Angiotensin II (1 uM) **(D)**. Representative data from one of 4 experiments are shown. **(E)** Relative fold change in SNAIL1-CBR bioluminescence intensity in cardiac fibroblasts exposed to increasing collagen I concentration. In each condition, values at t = 0 were arbitrarily set to equal 1. **(F)** SNAIL1-CBR bioluminescence intensity in cardiac fibroblasts plated on 4mg/mL collagen I for increasing lengths of time. A representative example from 3 experiments is shown. **(G)** Relative SNAIL1 mRNA fold change in primary cardiac fibroblasts 4 hours post stimulation with indicated factors, as determined by Q-PCR. All values were normalized to GAPDH and compared to non-treated cells as control. SNAIL1 mRNA level in nontreated cells was arbitrarily set to 1. Representative example of one of 3 experiments is shown.

Following myocardial infarction a number of pro-fibrotic cytokines including TGFβ and PDGF are released into the injured region[3]. To determine whether these pro-fibrotic factors affected SNAIL1 expression in cardiac fibroblasts/myofibroblasts, we isolated primary cardiac fibroblasts from normal, uninfarcted SNAIL1-CBR/+ mice. Their fibroblastic origin was confirmed by intermediate filament vimentin staining and Discoidin Domain Receptor 2 (DDR2) western blot which are both markers of cardiac fibroblasts [2](S3A and S3B Fig). Since SNAIL1 expression can be induced simply by exposing fibroblasts to serum[14], cardiac fibroblasts were first starved of serum for 4 hours prior to treating with pro-fibrotic factors. TGFβ, PDGF, CoCl_2_ (hypoxia mimetic agent)[19] and angiotensin II all increased SNAIL1-CBR bioluminescence signal (i.e., increased SNAIL1 protein) (Fig 3A–3D). In combination these factors did not exhibit any additive effect (S3C Fig).

Since the cardiac fibroblast marker DDR2 is a fibrillar collagen receptor that has been shown to stabilize and activate SNAIL1 protein level[20], we asked whether the presence of collagen, that occurs post ischemic injury, could also induce or stabilize SNAIL1 expression. When SNAIL1-CBR primary cardiac fibroblasts were plated on increasing amounts of collagen I matrix and with increasing time, SNAIL1-CBR bioluminescence level increased (i.e., SNAIL1 protein level) (Fig 3E and 3F). Most of the effect of these pro-fibrogenic factors upon SNAIL1 level was at the protein level, as only TGFβ treatment increased SNAIL1 mRNA (Fig 3G).

### SNAIL1 is required for ECM collagen production and deposition by cardiac fibroblasts/myofibroblasts

**Fig 4:**
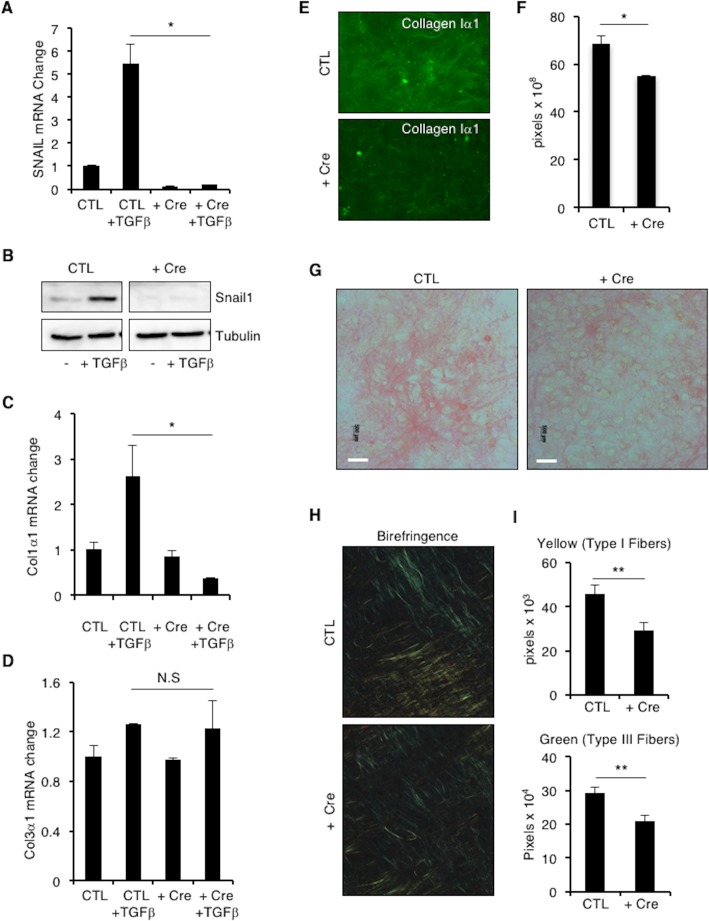
**SNAIL1-deleted cardiac fibroblasts have decreased collagen I mRNA expression and collagen matrix deposition. (A)** Relative SNAIL1 mRNA level in isolated SNAIL1^*f/f*^; ROSA-LSL-tdTomato cardiac fibroblasts treated with Adeno-LacZ (CTL) or Adeno-Cre (+ Cre), with and without TGFβ stimulation (2ng/ml for 2 hours), as determined by Q-PCR. SNAIL1 mRNA level in CTL unstimulated cells was arbitrarily set to 1. **(B)** Western blot analysis, with the indicated antibodies, of extracts from cardiac fibroblasts isolated from SNAIL1^*f/f*^; ROSA-LSL-tdTomato mice and treated with Adeno-LacZ (CTL) or Adeno-Cre (+ Cre), with and without TGFβ stimulation (2 ng/ml for 2 hours). Col1α1 **(C)** and Col3α1 **(D)** mRNA levels at baseline and following TGFβ stimulation (2 ng/ml for 2 hours) in control (CTL) and SNAIL1^-/-^ (+ Cre) cardiac fibroblasts. Shown is a representative result of one of 4 independent experiments. **(E)** Collagen I immunofluorescence of cell free matrix deposited by control (CTL) and SNAIL1^-/-^ (+ Cre) cardiac fibroblasts. (n = 3). **(F)** Quantification of results in (E). **(G)** Detection of Collagen 1 by Sirius Red Staining of cell free matrix deposited by control (CTL) and SNAIL1-deleted (+ Cre) cardiac fibroblasts. (n = 4). **(H)** Birefringence imaging of Sirius red stained matrix produced by control (CTL) or SNAIL^-/-^ (+ Cre) cardiac fibroblasts. **(I)** Quantification of birefriengence imaging (n = 4, 20 images counted for each experiment). Scale bars: 50 microns.

As activated fibroblasts or myofibroblasts are the primary source of matrix secreted in zones of ischemic injury[21], we asked whether SNAIL1 was necessary for matrix deposition by myofibroblasts. To do so SNAIL1^*f/f*^ mice[7] were crossed to ROSA26-LSL-tdTomato reporter mice. We did not observe any developmental or physiologic abnormality in SNAIL1 ^*f/f*^; ROSA26-LSL-tdTomato mice. Primary cardiac fibroblasts were isolated from 8 week old SNAIL1^*f/f*^; ROSA-LSL-tdTomato mice. To delete SNAIL1, these cells were infected with either Adenovirus-LacZ (control) or Adenovirus-Cre (+ Cre) virus for 4 hours. Cells infected with Cre-expressing lentiviruses will delete the SNAIL1 gene and turn on the tdTomato expression. SNAIL1 deletion was verified by Q-PCR for SNAIL1 mRNA (Fig 4A) and western blot (Fig 4B), and indirectly through tdTomato expression (S4A Fig).

There was no significant difference in proliferation or migration of control versus SNAIL1 deleted cells. (S4B and S4E Fig). To determine whether SNAIL1^-/-^ cardiac fibroblasts cells respond normally to pro-fibrotic cytokines, we treated cells with PDGF for 20 minutes. We did not oberve any difference in ERK, STAT1, and AKT signaling in SNAIL1^-/-^ cardiac fibroblasts (S4C Fig). When treated with TGFβ, however, Collagen I mRNA expression increased in control cells but not in SNAIL1^-/-^ cells (Fig 4C). We did not observe any significant change in collagen III mRNA level (Fig 4D).

To assess ECM production by cardiac fibroblasts, we plated control and SNAIL1^-/-^ cells in the presence of ascorbic acid and PDGF for 7 days. Cells were removed and the cell free matrix stained with antibodies against collagen I or with Sirius red. Matrix produced by SNAIL1 deleted cells exhibited decreased collagen I staining, as determined by quantitative immunofluorescence (Fig 4E, quantified in Fig 4F), decreased Sirius red staining (Fig 4G), and decreased type I and type III collagen fiber birefringence (Fig 4H, quantified in Fig 4I). These results indicated that pro-fibrotic factor induced expression of SNAIL1in cardiac myofibroblasts was necessary for efficient extracellular matrix deposition.

### SNAIL1 is important for conversion of cardiac fibroblasts to activated myofibroblasts

**Fig 5:**
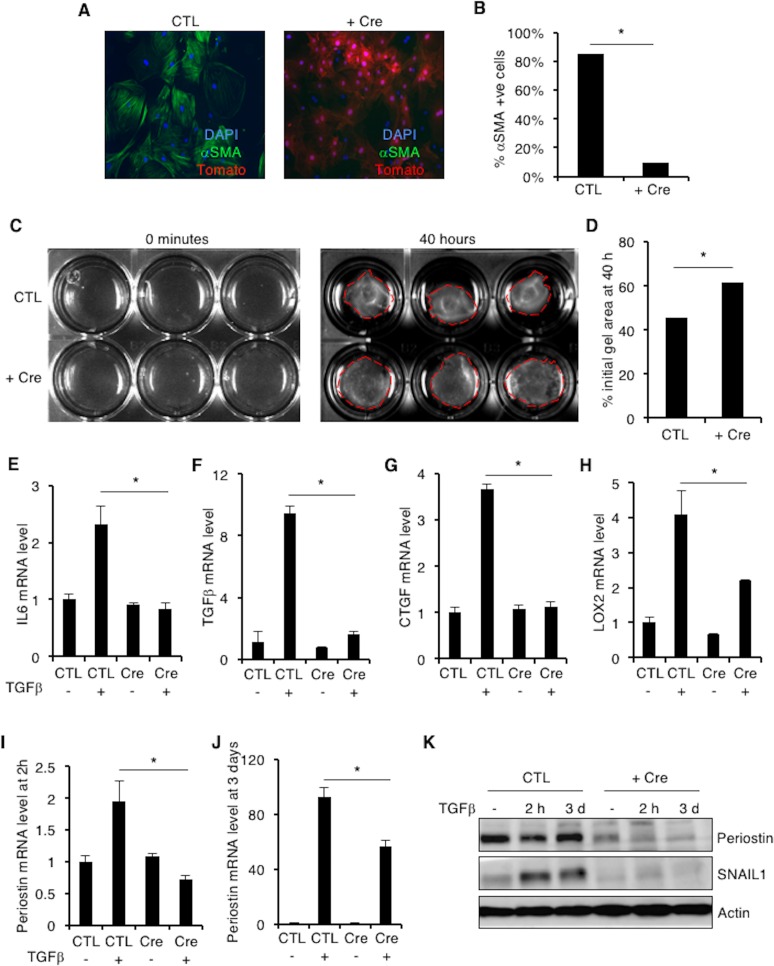
**SNAIL1-deleted cardiac fibroblasts have decreased αSMA, LoxL2 & Periostin expression. (A)** αSMA immunofluorescence of control (CTL) and SNAIL1-deleted (+ Cre) cardiac fibroblasts. **(B)** Quantification of results in (A). 30 fields were counted in each group. Shown is a representative result of one of 3 independent experiments. **(C)** Gel contraction assay of control (CTL) and SNAIL1^-/-^ (+ Cre) cardiac fibroblasts. **(D)** Quantification of (C). Shown is pooled data from 3 independent experiments, done in triplicates. **(E-J)** Relative fibrogenic gene mRNA expression in SNAIL1^-/-^ cardiac fibroblasts following TGFβ stimulation: IL6 (E), TGFβ (F), CTGF (G), LoxL2 (H), Periostin (I and J). Representative example of 3 separate experiments. **(K)** Western blot analysis with periostin antibody of extracts from control (CTL) and SNAIL1^-/-^ cardiac fibroblasts. Representative example of one of three separate experiments.

SNAIL1 action has been linked to cell fate changes. For example, during Epithelial Mesenchymal Transitions (EMT) and differentiation of embryonic stem (ES) cells[22]. Since SNAIL1 expression is induced as cardiac fibroblasts convert to myofibroblasts in response to pro-fibrotic factors or hypoxia, and SNAIL1 regulates matrix production by cardiac fibroblasts we asked whether SNAIL1 could be important for cardiac myofibroblast cell fate. To determine this, we isolated cardiac fibroblasts from SNAIL1^*f/f*^; ROSA-LSL-tdTomato mice and deleted SNAIL1 in these cells by infection with Adeno-Cre expressing viruses. Control cells were infected with Adeno-LacZ viruses. Cells were then activated by plating on plastic (high stiffness). Few αSMA positive (i.e., myofibroblast) cells (~10%) were detected in SNAIL1^-/-^ cultures (Fig 5A, quantified in Fig 5B). In this experiment, the presence of Tomato positive cells is a surrogate marker for SNAIL1gene deletion (i.e., both expression of Tomato and deletion of SNAIL1 are Cre responsive). Greater than 95% of Cre-infected cells were tomato positive (Fig 5A). This was in contrast to SNAIL1^+/+^ control cells. Control cells were tomato negative and ~85% of cells were αSMA positive (Fig 5A, quantified in Fig 5B).

The decrease in number of αSMA positive myofibroblasts in SNAIL1^-/-^ cultures was functionally relevant. SNAIL1^-/-^ cardiac fibroblasts were deficient in contracting collagen I gels (Fig 5C, quantified in Fig 5D). Furthermore, fibrotic gene expression (e.g., IL6, TGFβ and CTGF) and collagen remodeling gene expression (e.g., periostin and lysyl oxidase L2) were blunted in SNAIL1^-/-^ fibroblasts following exposure to TGFβ (Fig 5E–5J). Periostin protein level in SNAIL1^-/-^ fibroblasts was also decreased (Fig 5K). There was no difference in Lysyl oxidases L1 and L3 (S4D Fig). Collectively, these data suggested that SNAIL1 controls the adoption of the full myofibroblast phenotype by cardiac fibroblasts, at least following exposure to pro-fibrotic TGFβ and a stiff environment.

## Discussion

The wound healing response following a myocardial infarction (hypoxic injury) is necessary to form a scar in the infarcted region. Persistent deposition of extracellular matrix, predominantly collagens I and III, causes fibrosis in the infarct region, leading to decreased cardiac function. It is well appreciated that the cardiac myofibroblasts are responsible for collagen deposition in response to pro-fibrotic cytokines including TGFβ, PDGF and CTGF. SNAIL1, a zinc finger transcription factor, usually expressed in mesenchymal cells, is necessary for liver and kidney fibrosis in mice following injury using carbon tetrachloride and urinary ureter obstruction models respectively. In the heart, SNAIL1 has been shown to be expressed in cardiac fibroblasts and endothelial cells following Left Anterior Descending Artery Occlusion (LAD)[23] and ischemia-reperfusion (IR)[11] injury models, but whether SNAIL1 is necessary for fibrosis, especially in mesenchymal (non-epithelial) cells in the heart following injury and if so, the possible mechanism is not well understood.

The infarcted myocardium contains many cell lineages, including cardiac myofibroblasts, endothelial cells, circulating fibrocytes, bone marrow derived progenitor cells and monocytes[2]. Similar to previously reported studies, we observe elevated SNAIL1 levels 7 days post hypoxic injury by Left Anterior Descending Artery Occlusion[23] and show that SNAIL1 is expressed predominantly in myofibroblasts, as identified by αSMA and periostin staining, and a small proportion of CD45+ immune cells. About 40% of the myofibroblasts express SNAIL1. It is possible that SNAIL1 is expressed in only a subset of myofibroblasts as myofibroblasts in the heart can arise from different sources[2]. Alternatively, SNAIL1 has a short half-life of about 20 minutes and not all the myofibroblasts may be simultaneously expressing SNAIL1 maximally[24]. We do not find SNAIL1 expression in endothelial cells as previously reported[11]. It is possible that SNAIL1 is expressed in endothelial cells during early stages of wound healing, which might enable the endothelial cells to undergo endothelial to mesenchymal transition after cardiac injury[25] and contribute to increased number of myofibroblasts in the infarct area. The increase in SNAIL1 level correlates with increased SNAIL1 mRNA, collagen deposition in the infarct area and increased levels of pro-fibrotic cytokines. Interestingly, these pro-fibrotic cytokines can elevate SNAIL1 levels in cultured primary cardiac fibroblasts isolated from normal, uninjured hearts. Cardiac fibroblasts/myofibroblasts in the infarcted region express pro-fibrotic cytokines like TGFβ, CTGF and IL6 but SNAIL1-deleted cardiac fibroblasts do not increase pro-fibrotic cytokine gene expression in response to TGFβ and mechanical stress (culturing on high tension plastic tissue culture plates). This suggests that the presence of SNAIL1 in cardiac fibroblasts/myofibroblasts in the infarct region is responsible for increased the pro-fibrotic genes expression.

SNAIL1-CBR/+ mice had minimal SNAIL1 bioluminescence signal at baseline conditions. This was observed in multiple animals, even when the excised heart was cut open and imaged for longer time. The bioluminescence data is supported by immunofluorescence staining for SNAIL1 which is absent in hearts from sham surgery animals and remote zone of LAD surgery animals, but present in the infarct zone. This observation may result from the fact that SNAIL1 protein has an extremely short half-life (20 minutes in some cells), and external signals are required to maintain both mRNA and protein expression. Under stimulating conditions, for example exposure to TGFβ or hypoxia, cells elevate SNAIL1 mRNA and protein level. In the normal, un-injured heart, stimuli that increase SNAIL1 protein levels are absent (hypoxia, stiffness) or inactive (for example, TGFβ in latent form in the cardiac ECM). Thus when the heart (or cardiac fibroblasts) are injured, signals that increase SNAIL1 protein levels are elevated.

Collagens I and III are the primary collagens secreted by the cardiac fibroblasts/myofibroblasts in the infarcted myocardium in response to pro-fibrotic factors. SNAIL1-deleted cardiac fibroblasts have reduced collagen I mRNA expression and collagen deposition in response to TGFβ. Collagen III expression in response to TGFβ was unaffected by the absence of SNAIL1. This may reflect distinct regulation of Collagen I and III gene expression in cardiac fibroblasts, since collagen III but not collagen I in cardiac fibroblasts is regulated by mechanical stretch signals[26]. Moreover, post myocardial infarction, Collagen III is laid down initially which stabilizes the scar and over time Collagen I replaces Collagen III[27]. Additionally, SNAIL1 is necessary for extracellular collagen modifying and crosslinking genes expression, i.e. LoxL2 and periostin. This data suggests that SNAIL1 is important for collagen deposition and remodeling. Since increased collagen I matrix increases SNAIL1 levels in cardiac fibroblasts this suggests a feed forward loop whereby SNAIL1 causes increased collagen deposition, which in turn maintains elevated SNAIL1 in the cardiac fibroblasts, possibly through the collagen receptor DDR2[20] or mechanical signals through Integrin activation[9]

The cardiac fibroblasts adopt a myofibroblast fate during fibrosis and increase in number in the infarcted region. SNAIL1 expression is responsible for cell fate changes, for example, during Epithelial to Mesenchymal transition[13] and during embryonic stem cell differentiation[22]. Whether SNAIL1 is responsible for the transformation of cardiac fibroblast to myofibroblasts is unknown. We show that by knocking out SNAIL1, the cardiac fibroblasts do not express the myofibroblast marker αSMA. It is possible that through activation of Rho kinase, the myocardin related transcription factor (MRTF), which can mediate TGFβ induced EMT[28] and also αSMA expression[29], might be either interacting / affecting the activity of SNAIL1 and vice versa.

TGFβ treatment of cardiac fibroblasts upregulates multiple genes including ECM genes (collagens), ECM modifiers (periostin, LoxL2) and other profibrotic cytokines (IL6, CTGF). SNAIL1 gene transcription and protein levels are also increased following TGFβ yet SNAIL1 is a transcriptional repressor. So how does SNAIL1 deletion abrogate the elevation of the above mentoned TGFβ, target genes? One possibility is that SNAIL1 could be acetylated in cardiac fibroblasts under these conditions, and as such functions as a transcriptional (co)activator[12]. Alternatively, TGFβ could repress microRNA29 via SNAIL1 action, which then releases miR29’s inhibition of collagen I and III expression[30].

In sum, our data suggest that cardiac fibroblasts in the infarcted myocardium express SNAIL1 and adopt a myofibroblast phenotype. SNAIL1 contributes to fibrosis by allowing expression of pro-fibrotic cytokines and deposition and remodeling of the collagen matrix causing a scar. The pro-fibrotic cytokines and collagen I in turn maintain SNAIL1 levels in the myofibroblasts to sustain the fibrotic state. It remains to be determined whether SNAIL1 deletion in cardiac fibroblasts in-vivo reduces fibrosis post myocardial infarction in mice. To enable this study, SNAIL1^*f/f*^; Rosa LSL tdTomato mice can be crossed to Periostin-Cre which is expressed in cardiac fibroblasts post myocardial injury[31].

## Supporting Information

S1 Fig SNAIL1 expression in the heart pre- and post-ischemic injury. (TIFF)

S2 Fig Histologic expression of cardiac cell markers in non-infarcted hearts. (TIFF)

S3 Fig Characterization of cultured primary cardiac cells. (TIFF)

S4 Fig Characterization of SNAIL1^-/-^ cardiac fibroblasts. (TIFF)
