# Supplementary figures and images for: Action of SNAIL1 in Cardiac Myofibroblasts Is Important for Cardiac Fibrosis following Hypoxic Injury
Source: PLoS One. 2016 Oct 5;11(10):e0162636. doi: 10.1371/journal.pone.0162636 (PMC5051686; doi:10.1371/journal.pone.0162636)

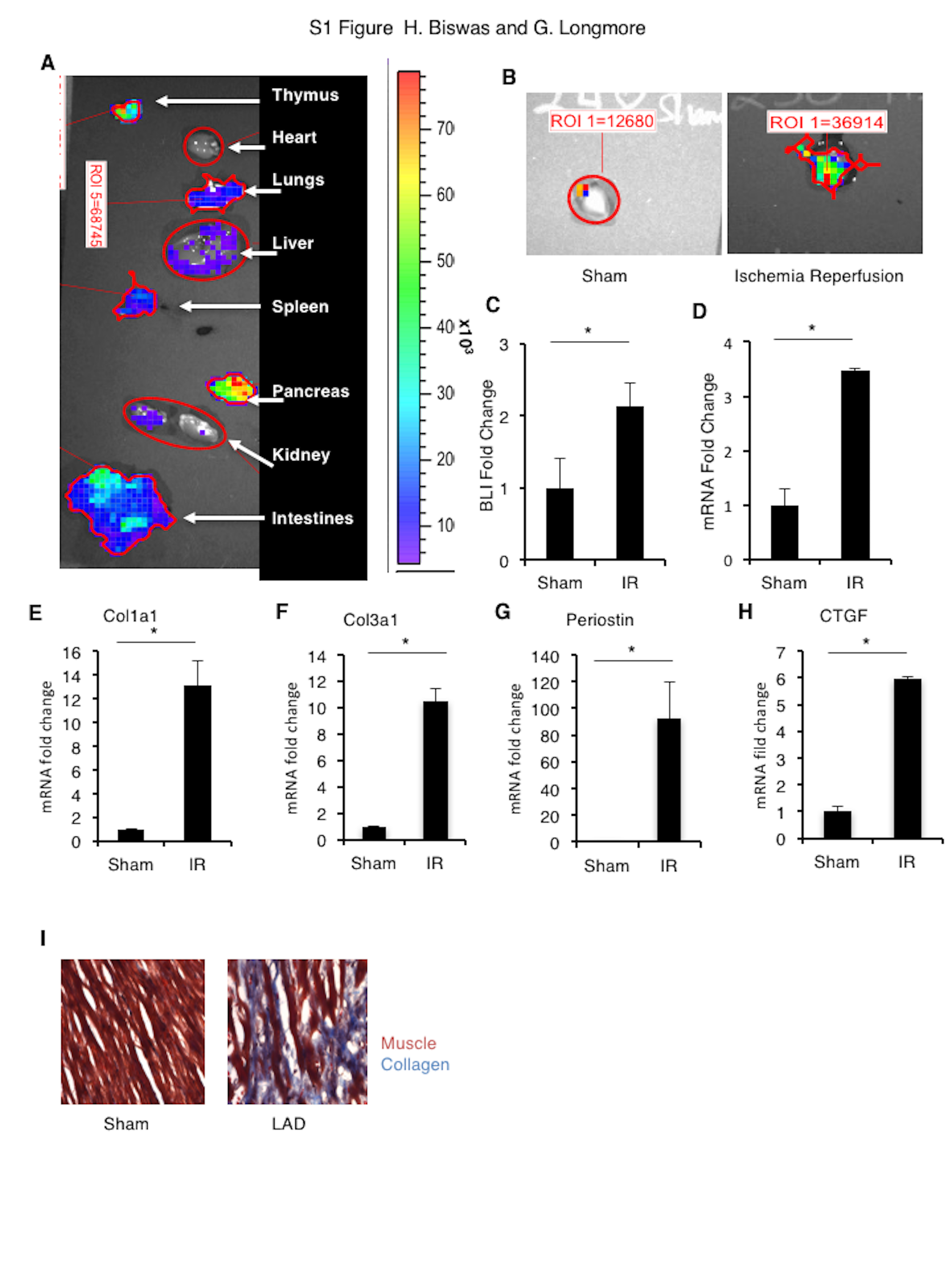

Supplement: S1 Fig — (TIFF) [file pone.0162636.s001.tiff]

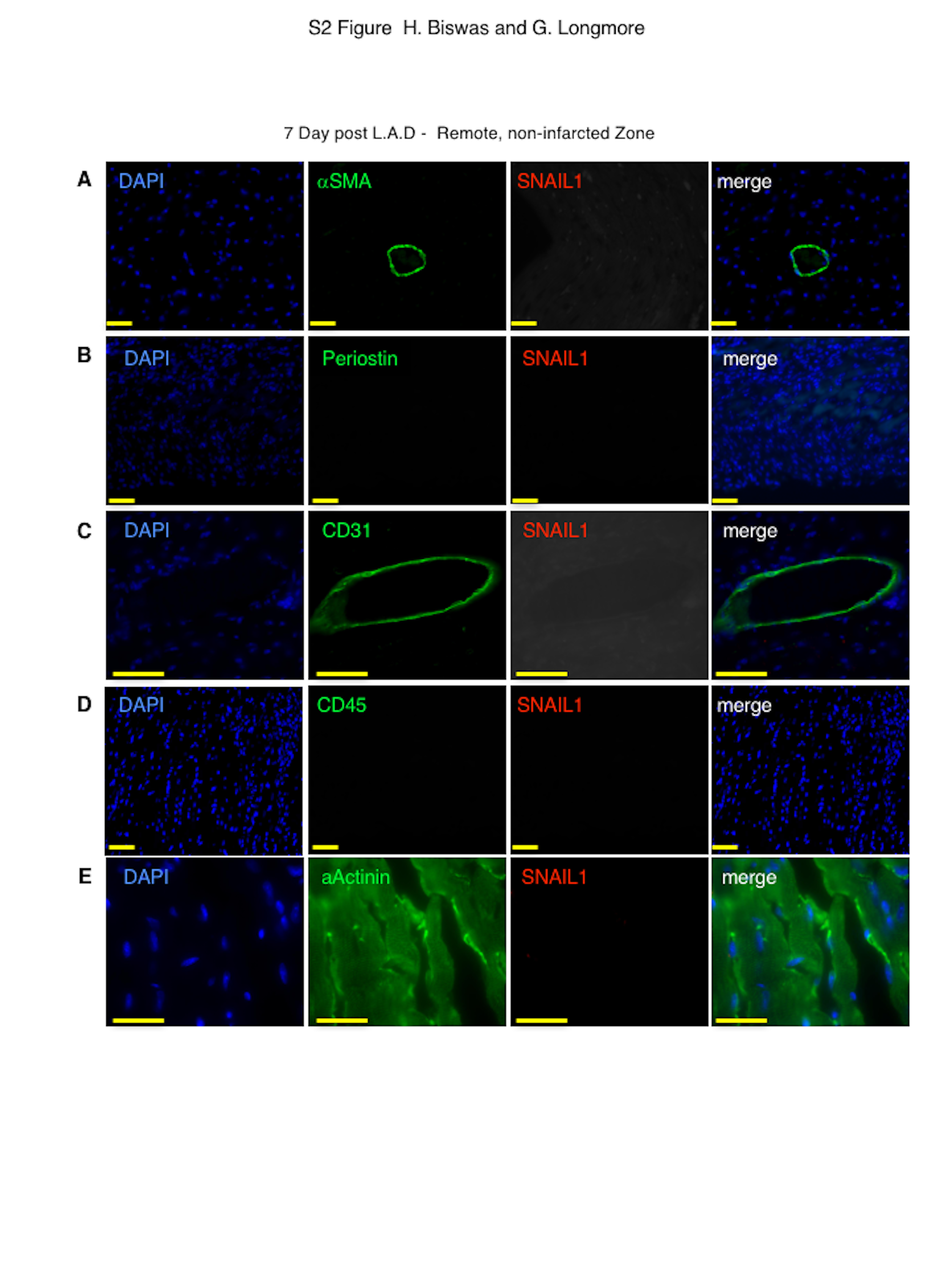

Supplement: S2 Fig — (TIFF) [file pone.0162636.s002.tiff]

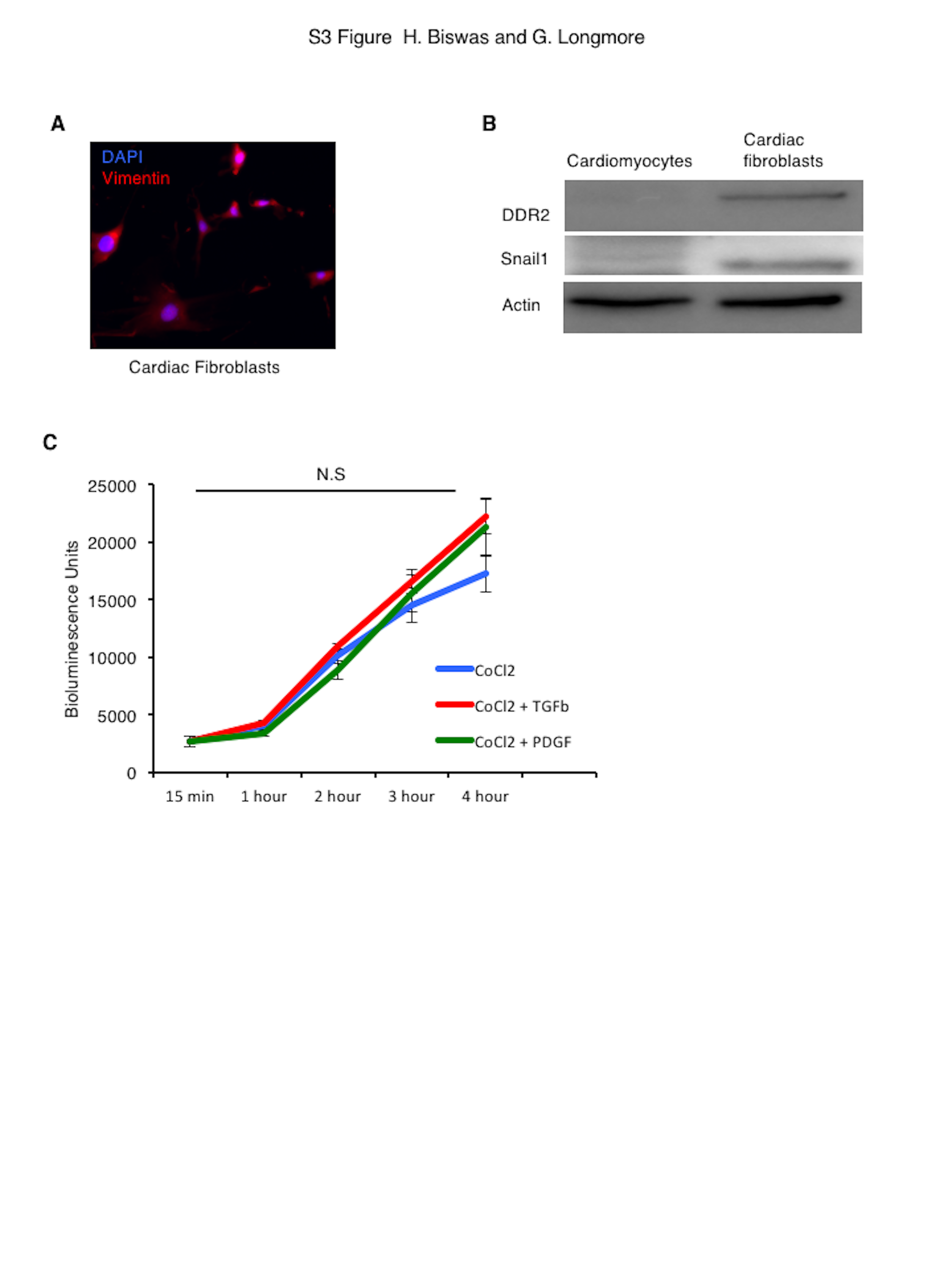

Supplement: S3 Fig — (TIFF) [file pone.0162636.s003.tiff]

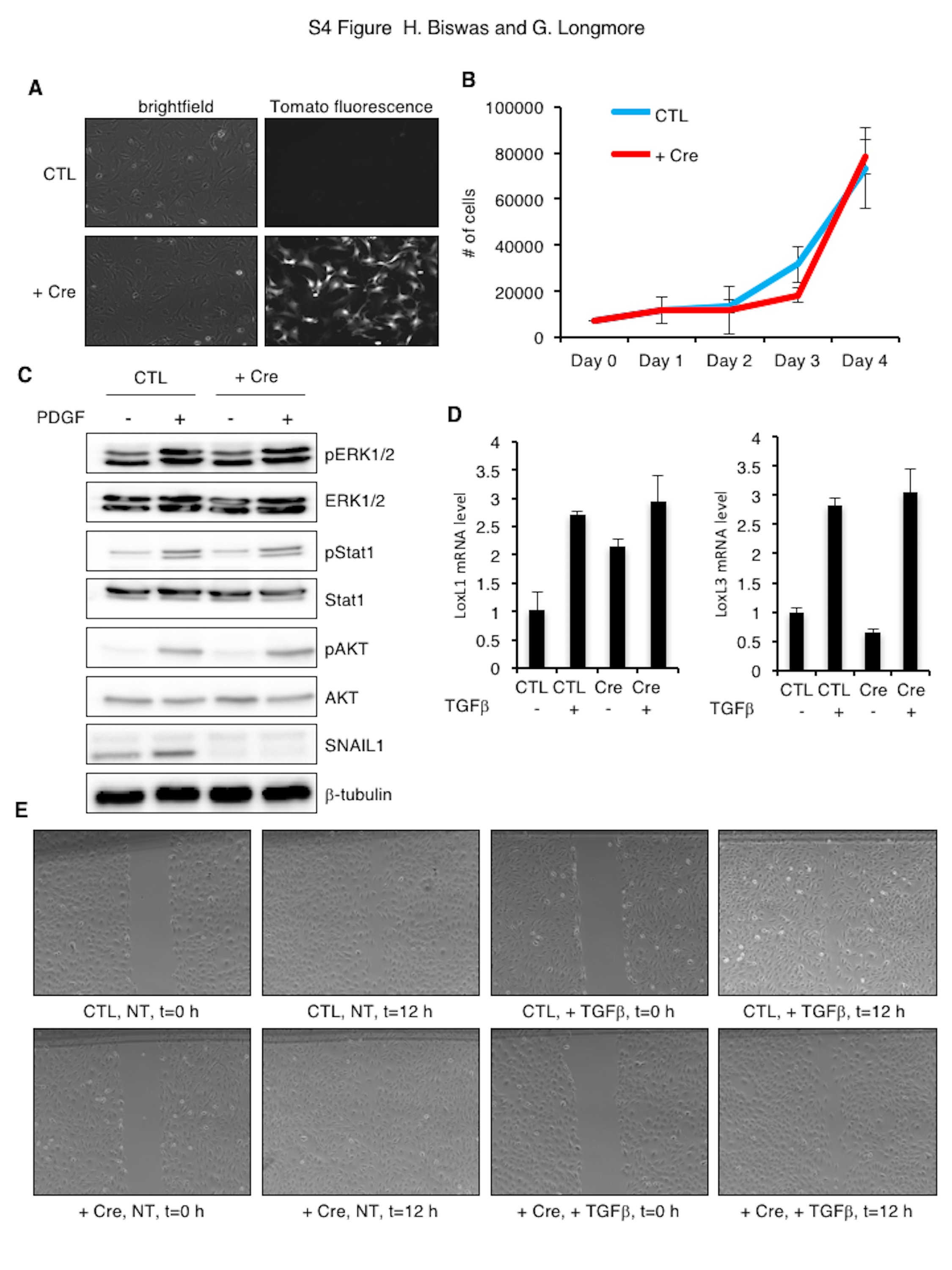

Supplement: S4 Fig — (TIFF) [file pone.0162636.s004.tiff]
